# Supplementary material for: High-Impact Risk Factors for Mycobacterium avium ssp. paratuberculosis in Dairy Herds in Germany
Source: Animals (Basel). 2023 Jun 6;13(12):1889. doi: 10.3390/ani13121889 (PMC10294860; doi:10.3390/ani13121889)
Supplement: Supplementary file 1 [file animals-13-01889-s001.zip › animals-2412765-supplementary.pdf]

Supplementary Material

High-Impact Risk Factors for *Mycobacterium avium* ssp. *paratuberculosis* in Dairy Herds in Germany

Mette Krieger *, Susanne Eisenberg, Karsten Donat and Amely Campe

*** Correspondence:** Mette.Krieger@tiho-hannover.de

1 Supplementary Tables

Supplementary Table S1: Descriptive and single-factorial analyses of collected categorical variables considered to be risk factors for MAP-positive dairy herds (*Mycobacterium avium* ssp. *paratuberculosis* had been isolated from at least one environmental sample)

**Variable**	**Category**	**MAP-positive herds n (%)**	**MAP-negative herds n (%)**	**Herds with information available n (%)**	**OR**	**LCL**	**UCL**	**p**	**AFe**	**AFp**
Herd characteristics										
Breed	80% Holstein	N: 18 (81.82)<br>E: 56 (81.16)<br>S: 0 (0.0)	N: 138 (85.71)<br>E: 71 (70.30)<br>S: 1 (0.99)	N: 183 (100)<br>E: 170 (100)<br>S: 104 (100)	N, E: No logistic regression conducted due to missing variation					
	80% Simmental cattle	N: 0 (0.0)<br>E: 1 (1.45)<br>S: 2 (66.67)	N: 0 (0.0)<br>E: 1 (0.99)<br>S: 82 (81.19)						N: n.p.<br>E: 0.12	N: 0.00<br>E: 0.00
	80% Other	N: 4 (18.18)<br>E: 12 (17.39)<br>S: 1 (33.33)	N: 23 (14.29)<br>E: 29 (28.71)<br>S: 18 (17.82)						N: 0.22<br>E: -0.51	N: 0.04<br>E: -0.09
Housing	Tiestall	N: 0 (0.0)<br>E: 0 (0.0)<br>S: 0 (0.0)	N: 7 (4.35)<br>E: 3 (2.97)<br>S: 33 (32.67)	N: 161 (87.98)<br>E: 132 (77.65)<br>S: 101 (97.12)	N, E: No logistic regression possible due to missing variation					
	Freestall	N: 21 (95.45)<br>E: 56 (81.16)<br>S: 3 (100)	N: 133 (82.61)<br>E: 73 (72.28)<br>S: 65 (64.36)						N: 1.00<br>E: 1.00	N: 1.00<br>E: 1.00
General farm management: External biosecurity										
Did you purchase cattle in the last 12 months?	No[1]	N: 12 (54.55)<br>E: 32 (46.38)<br>S: 2 (66.67)	N: 90 (55.90)<br>E: 68 (67.33)<br>S: 75 (74.26)	N: 182 (99.45)<br>E: 170 (100)<br>S: 104 (100)				N: 0.34[a]<br>E: 0.01[a]		
	Yes with inquiring about the MAP status	N: 5 (22.73)<br>E: 6 (8.70)<br>S: 0 (0.0)	N: 19 (11.80)<br>E: 10 (9.90)<br>S: 2 (1.98)		N: 1.97<br>E:1.28	N: 0.62<br>E: 0.43	N: 6.26<br>E: 3.81	N: 0.15<br>E: 0.60	N: 0.44<br>E: 0.15	N: 0.13<br>E: 0.22
	Yes without inquiring about the MAP status	N: 5 (22.73)<br>E: 31 (44.93)<br>S: 1 (33.33)	N: 51 (31.68)<br>E: 23 (22.77)<br>S: 24 (23.76)		N: 0.74<br>E: 2.86	N: 0.25<br>E: 1.45	N: 2.21<br>E: 5.67	N: 0.24<br>E: 0.02	N: -0.32<br>E: 0.44	N: -0.09<br>E: 0.02

Variable	Category	MAP-positive herds n (%)	MAP-negative herds n (%)	Herds with information available n (%)	OR	LCL	UCL	p	AFe	AFp
Is cattle that has left the herd reintegrated directly into the herd?	No[1]	N: 1 (4.55)<br>E: 7 (10.14)<br>S: 1 (33.33)	N: 8 (4.97)<br>E: 9 (8.91)<br>S: 23 (22.77)	N: 47 (25.68)<br>E: 57 (33.53)<br>S: 45 (43.27)						
	Yes	N: 4 (18.18)<br>E: 21 (30.43)<br>S: 0 (0.0)	N: 34 (21.12)<br>E: 20 (19.80)<br>S: 21 (20.79)		N: 0.94<br>E: 1.35	N: 0.09<br>E: 0.42	N: 9.6<br>E: 4.32	N: 0.96<br>E: 0.61	N: -0.06<br>E: 0.15	N: -0.04<br>E: 0.11
Do you share equipment or vehicles that is used in livestock farming with other farms (e.g., cattle trucks, hoof trimming chutes...)?	No[1]	N: 20 (90.91)<br>E: 66 (95.65)<br>S: 2 (66.67)	N: 128 (79.50)<br>E: 89 (88.12)<br>S: 70 (69.31)	N: 182 (99.45)<br>E: 170 (100)<br>S: 103 (99.04)						
	Yes	N: 2 (9.09)<br>E: 3 (4.35)<br>S: 1 (33.33)	N: 32 (19.88)<br>E: 12 (11.88)<br>S: 30 (29.70)		N: 0.4<br>E: 0.34	N: 0.09<br>E: 0.09	N: 1.8<br>E: 1.24	N: 0.23<br>E: 0.1	N: -1.30<br>E: -1.13	N: -0.12<br>E: -0.05
Are you keeping young cattle*[1] on pasture?	No[1]	N: 6 (27.27)<br>E: 39 (56.52)<br>S: 3 (100)	N: 49 (30.43)<br>E: 53 (52.48)<br>S: 58 (57.43)	N: 183 (100)<br>E: 170 (100)<br>S: 104 (100)						
	Yes	N: 16 (72.73)<br>E: 30 (43.48)<br>S: 0 (0.0)	N: 112 (69.57)<br>E: 48 (47.52)<br>S: 43 (42.57)		N: 1.17<br>E: 0.8	N: 0.43<br>E: 0.43	N: 3.16<br>E: 1.48	N: 0.76<br>E: 0.48	N: 0.13<br>E: -0.10	N: 0.09<br>E: -0-04
Is it possible to clean boots and hands before leaving each location of the farm?	No	N: 9 (40.91)<br>E: 5 (7.25)<br>S: 0 (0.0)	N: 46 (28.57)<br>E: 8 (7.92)<br>S: 17 (16.83)	N: 172 (93.99)<br>E: 168 (98,82)<br>S: 103 (99.04)						
	Yes[1]	N: 12 (54.55)<br>E: 63 (91.30)<br>S: 3 (100)	N: 105 (65.22)<br>E: 92 (91.09)<br>S: 83 (82.18)		N: 1.71<br>E: 0.91	N: 0.68<br>E: 0.29	N: 4.34<br>E: 2.92	N: 0.26<br>E: 0.88	N: 0.37<br>E: -0.06	N: 0.16<br>E: 0.00
What kind of livestock farming (besides dairy cow farming) is here on	No[1]	N: 19 (86.36)<br>E: 44 (63.77)<br>S: 3 (100)	N: 113 (70.19)<br>E: 62 (61.39)<br>S: 79 (78.22)	N: 183 (100)<br>E: 170 (100)<br>S: 104 (100)						
	Yes	N: 3 (13.64)	N: 48 (29.81)		N: 0.37	N: 0.11	N: 1.32	N: 0.13	N: -1.45	N: -0.20

Variable	Category	MAP-positive herds n (%)	MAP-negative herds n (%)	Herds with information available n (%)	OR	LCL	UCL	p	AFe	AFp
the farm?: Game animals, goats/sheep, pigs, horses (breeding, fattening, pension), poultry, water fowl		E: 25 (36.23)<br>S: 0 (0.0)	E: 39 (38.61)<br>S: 22 (21.78)		E: 0.9	E: 0.5	E: 1.7	E: 0.75	E: -0.06	E: -0.02
General farm management: Internal biosecurity										
Is it possible to clean boots and hands before leaving each stable of the farm?	No	N: 20 (90.91)<br>E: 64 (92.75)<br>S: 2 (66.67)	N: 142 (88.20)<br>E: 90 (89.11)<br>S: 45 (44.55)	N: 171 (93.44)<br>E: 167 (98.24)<br>S: 103 (99.04)						
	Yes[1]	N: 1 (4.55)<br>E: 4 (5.80)<br>S: 1 (33.33)	N: 8 (4.97)<br>E: 9 (8.91)<br>S: 55 (54.46)		N: 1.13<br>E: 1.6	N: 0.13<br>E: 0.47	N: 9.49<br>E: 5.42	N: 0.91<br>E: 0.45	N: 0.10<br>E: 0.26	N: 0.10<br>E: 0.24
Fecal contamination of the lying areas	Clean or single excrements on lying area[1]	N: 5 (22.73)<br>E: 20 (28.99)<br>S: 2 (66.67)	N: 44 (27.33)<br>E: 38 (37.62)<br>S: 29 (28.71)	N: 180 (98.36)<br>E: 168 (98.82)<br>S: 102 (98.08)				N: 0.57[a]<br>E: 0.38[a]		
	<50%	N: 16 (72.73)<br>E: 47 (68.12)<br>S: 1 (33.33)	N: 98 (60.87)<br>E: 54 (53.47)<br>S: 61 (60.40)		N: 1.44<br>E: 1.65	N: 0.5<br>E: 0.85	N: 4.17<br>E: 3.23	N: 0.29<br>E: 0.96	N: 0.27<br>E: 0.26	N: 0.21<br>E: 0.18
	>50%, but surface still visible	N: 1 (4.55)<br>E: 2 (2.90)<br>S: 0 (0.0)	N: 16 (9.94)<br>E: 6 (5.94)<br>S: 8 (7.92)		N: 0.55<br>E: 0.63	N: 0.06<br>E: 0.12	N: 5.07<br>E: 3.43	N: 0.47<br>E: 0.98	N: -0.73<br>E: -0.38	N: -0.12<br>E: -0.03
	Completely covered with feces	N: 0 (0.0)<br>E: 0 (0.0)<br>S: 0 (0.0)	N: 0 (0.0)<br>E: 1 (0.99)<br>S: 1 (0.99)		N, E: No logistic regression possible due to missing variation				N: n.p.<br>E: n.p.	N: 0.00<br>E: -0.02
Fecal contamination of the walkways	Clean or single excrements on walk way[1]	N: 3 (13.64)<br>E: 11 (15.94)<br>S: 1 (33.33)	N: 21 (13.04)<br>E: 12 (11.88)<br>S: 6 (5.94)	N: 175 (95.63)<br>E: 158 (92.94)<br>S: 89 (85.58)				N: 0.95[a]<br>E: 0.14[a]		
	<50%	N: 13 (59.09)<br>E: 47 (68.12)	N: 97 (60.25)<br>E: 53 (52.48)		N: 0.94<br>E: 0.97	N: 0.25<br>E: 0.39	N: 3.59<br>E: 2.4	N: 0.98<br>E: 0.93	N: -0.06<br>E: -0.02	N: -0.05<br>E: -0.01

Variable	Category	MAP-positive herds n (%)	MAP-negative herds n (%)	Herds with information available n (%)	OR	LCL	UCL	p	AFe	AFp
		S: 2 (66.67)	S: 47 (46.53)							
	>50%, but surface still visible	N: 6 (27.27)<br>E: 8 (11.59)<br>S: 0 (0.0)	N: 33 (20.50)<br>E: 24 (23.76)<br>S: 33 (32.67)		N: 1.27<br>E: 0.36	N: 0.29<br>E: 0.12	N: 5.65<br>E: 1.14	N: 0.97<br>E: 0.04	N: 0.19<br>E: -0.91	N: 0.13<br>E: -0.38
	Completely covered with feces	N: 0 (0.0)<br>E: 2 (2.90)<br>S: 0 (0.0)	N: 2 (1.24)<br>E: 1 (0.99)<br>S: 0 (0.0)		N: <0.001<br>E: 2.18	N:<0.001<br>E: 0.17	N:>999.9<br>E: 27.56	N: 0.97<br>E: 0.36	N: n.p.<br>E: 0.28	N: -0.08<br>E: 0.04
Calf management: Calving										
Where did the most calvings take place within the last 12 months?	Calving only in individual calving pen [1]	N: 6 (27.27)<br>E: 10 (14.49)<br>S: 1 (33.33)	N: 40 (24.84)<br>E: 13 (12.87)<br>S: 11 (10.89)	N: 183 (100)<br>E: 170 (100)<br>S: 104 (100)				N: 0.98[a]<br>E: 0.96[a]		
	Calving only in common stabling	N: 2 (9.09)<br>E: 3 (4.35)<br>S: 0 (0.0)	N: 8 (4.97)<br>E: 6 (5.94)<br>S: 46 (45.54)		N: 1.67<br>E: 0.65	N: 0.28<br>E: 0.13	N: 9.8<br>E: 3.26	N: 0.96<br>E: 0.97	N: 0.35<br>E: -0.30	N: 0.09<br>E: -0.07
	Calving only in group calving pen	N: 8 (36.36)<br>E: 48 (69.57)<br>S: 2 (66.67)	N: 59 (36.65)<br>E: 63 (62.38)<br>S: 16 (15.84)		N: 0.9<br>E: 0.99	N: 0.29<br>E: 0.4	N: 2.8<br>E: 2.45	N: 0.97<br>E: 0.97	N: -0.09<br>E: -0.01	N: -0.05<br>E: 0.00
	Calving only in pen used for sick animals	N: 3 (13.64)<br>E: 2 (2.9)<br>S: 0 (0.0)	N: 28 (17.39)<br>E: 2 (1.98)<br>S: 14 813.86)		N: 0.71<br>E: 1.3	N: 0.17<br>E: 0.16	N: 3.1<br>E: 10.9	N: 0.97<br>E: 0.96	N: -0.35<br>E: 0.13	N: -0.12<br>E: 0.02
	Calving only on pasture	N: 0 (0.0)<br>E: 0 (0.0)<br>S: 0 (0.0)	N: 8 (4.97)<br>E: 5 (4.95)<br>S: 1 (0.99)		N, E: No logistic regression possible due to missing variation				N: n.p.<br>E: n.p.	N: -0.17<br>E: -0.22
	Calving only in another area	N: 0 (0.0)<br>E: 0 (0.0)<br>S: 0 (0.0)	N: 0 (0.0)<br>E: 0 (0.0)<br>S: 5 (4.95)		N, E: No logistic regression possible due to missing variation				N: n.p.<br>E: n.p.	N: 0.00<br>E: 0.00
	Calving in combination of above areas	N: 3 (13.64)<br>E: 6 (8.70)<br>S: 0 (0.0)	N: 18 (11.18)<br>E: 12 (11.88)<br>S: 8 (7.92)		N: 1.11<br>E: 0.65	N: 0.25<br>E: 0.18	N: 4.95<br>E: 2.34	N: 0.96<br>E: 0.97	N: 0.09<br>E: -0.30	N: 0.03<br>E: -0.11

Variable	Category	MAP-positive herds n (%)	MAP-negative herds n (%)	Herds with information available n (%)	OR	LCL	UCL	p	AFe	AFp
Does the calf always stay with the mother for a certain time after birth? (suckling calves)	No[1]	N: 10 (45.45)<br>E: 38 (55.07)<br>S: 2 (66.67)	N: 67 (41.61)<br>E: 42 (41.58)<br>S: 69 (68.32)	N: 183 (100)<br>E: 168 (98.82)<br>S: 104 (100)						
	Yes	N: 12 (54.54)<br>E: 30 (43.48)<br>S: 1 (33.33)	N: 94 (58.38)<br>E: 58 (57.42)<br>S: 32 (31.68)		N: 0.86<br>E: 0.57	N: 0.35<br>E: 0.31	N: 2.1<br>E: 1.07	N: 0.73<br>E: 0.08	N: -0.15<br>E: -0.39	N: -0.08<br>E: -0.17
Calf management: Calf housing										
Which hygiene measures are conducted before stabling new calves?	Hygiene measures with low risk of infection[1] †	N: 21 (95.45)<br>E: 66 (95.65)<br>S: 3 (100.00)	N: 125 (77.64)<br>E: 89 (88.12)<br>S: 92 (91.09)	N: 163 (89.07)<br>E: 159 (93.53)<br>S: 101 (97.12)				N: 0.99[a]<br>E: 0.77[a]		
	Hygiene measures with high risk of infection ‡	N: 0 (0.0)<br>E: 2 (2.9)<br>S: 0 (0.0)	N: 11 (6.83)<br>E: 2 (1.98)<br>S: 5 (4.95)		N: <0.001<br>E: 1.35	N:<0.001<br>E: 0.19	N:>999.9<br>E: 9.82	N: 0.98<br>E: 0.77	N: n.p.<br>E: 0.15	N: -0.08<br>E: 0.00
	Other hygiene measures	N: 0 (0.0)<br>E: 0 (0.0)<br>S: 0 (0.0)	N: 6 (3.73)<br>E: 0 (0.0)<br>S: 1 (0.99)		N, E: No logistic regression possible due to missing variation				N: n.p.<br>E: n.p.	N: -0.04<br>E: 0.00
Are calves up to two weeks housed in group housing?	No[1]	N: 21 (95.45)<br>E: 63 (91.30)<br>S: 3 (100)	N: 151 (93.79)<br>E: 84 (83.17)<br>S: 100 (99.01)	N: 183 (100)<br>E: 170 (100)<br>S: 104 (100)						
	Yes	N: 1 (4.55)<br>E: 6 (8.70)<br>S: 0 (0.0)	N: 10 (6.21)<br>E: 17 (16.83)<br>S: 1 (0.99)		N: 0.72<br>E: 0.47	N: 0.09<br>E: 0.18	N: 5.91<br>E: 1.26	N: 0.76<br>E: 0.13	N: -0.34<br>E: -0.64	N: -0.02<br>E: -0.06
Are calves from three weeks on housed in group housing?	No[1]	N: 0 (0.0)<br>E: 1 (1.45)<br>S: 0 (0.0)	N: 3 (1.86)<br>E: 3 (2.97)<br>S: 8 (7.92)	N: 178 (97.27)<br>E: 168 (98.82)<br>S: 103 (99.04)						
	Yes	N: 20 (90.91)<br>E: 68 (98.55)<br>S: 3 (100)	N: 155 (96.27)<br>E: 96 (95.05)<br>S: 92 (91.09)		N: >999.9<br>E: 2.13	N:<0.001<br>E: 0.22	N:>999.9<br>E: 20.87	N: 0.99<br>E: 0.52	N: 1.00<br>E: 0.40	N: 1.00<br>E: 0.39

Variable	Category	MAP-positive herds n (%)	MAP-negative herds n (%)	Herds with information available n (%)	OR	LCL	UCL	p	AFe	AFp
Do the calves*[2] have contact with other calves if housing in an individual calf igloo?	No[1]	N: 5 (22.73)<br>E: 12 (17.39)<br>S: 1 (33.33)	N: 21 (13.04)<br>E: 17 (16.83)<br>S: 9 (8.91)	N: 77 (42.08)<br>E: 87 (51.18)<br>S: 24 (23.08)						
	Yes	N: 7 (31.82)<br>E: 31 (44.93)<br>S: 0 (0.0)	N: 44 (27.33)<br>E: 27 (26.73)<br>S: 14 (13.86)		N: 0.67<br>E: 1.63	N: 0.19<br>E: 0.66	N: 2.36<br>E: 4.01	N: 0.53<br>E: 0.29	N: -0.40<br>E: 0.23	N: -0.23<br>E: 0.16
Fecal contamination of the lying areas	Clean or single excrements on lying area[1]	N: 16 (72.73)<br>E: 41 (59.42)<br>S: 3 (100)	N: 110 (68.32)<br>E: 67 (66.34)<br>S: 73 (72.38)	N: 176 (96.17)<br>E: 166 (97.65)<br>S: 100 (96.15)				N: 0.85[a]<br>E: 0.44[a]		
	<50%	N: 5 (22.73)<br>E: 25 (36.23)<br>S: 0 (0)	N: 40 (24.84)<br>E: 27 (26.73)<br>S: 22 (21.78)		N: 0.86<br>E: 1.51	N: 0.3<br>E: 0.78	N: 2.5<br>E: 2.95	N: 0.57<br>E: 0.74	N: -0.14<br>E: 0.21	N: -0.03<br>E: 0.08
	>50%, but surface still visible	N: 1 (4.55)<br>E: 3 (4.35)<br>S: 0 (0)	N: 4 (2.48)<br>E: 3 (2.97)<br>S: 2 (1.98)		N: 1.72<br>E: 1.63	N: 0.18<br>E: 0.32	N: 16.36<br>E: 8.48	N: 0.59<br>E: 0.73	N: 0.37<br>E: 0.24	N: 0.02<br>E: 0.02
Do you practice calf rearing in addition to dairy cow farming on the farm?	Only rearing own calves[1]	N: 19 (86.36)<br>E: 63 (91.30)<br>S: 3 (100)	N: 140 (86.96)<br>E: 99 (98.02)<br>S: 96 (95.05)	N: 183 (100)<br>E: 170 (100)<br>S: 104 (100)				N: 0.24[a]<br>E: 0.17[a]		
	Rearing own calves and calves from other farms	N: 1 (4.55)<br>E: 5 (7.25)<br>S: 0 (0.0)	N: 17 (10.56)<br>E: 1 (0.99)<br>S: 3 (2.97)		N: 0.43<br>E: 7.86	N: 0.06<br>E: 0.9	N: 3.45<br>E: 68.82	N: 0.19<br>E: 0.16	N: -1.15<br>E: 0.53	N: -0.06<br>E: 0.04
	No calf rearing	N: 2 (9.09)<br>E: 1 (1.45)<br>S: 0 (0.0)	N: 4 (2.48)<br>E: 1 (0.99)<br>S: 2 (1.98)		N: 3.69<br>E: 1.57	N: 0.63<br>E: 0.1	N: 21.5<br>E: 25.58	N: 0.09<br>E: 0.70	N: 0.64<br>E: 0.22	N: 0.06<br>E: 0.00
Calf management: Calf feeding										
Do you pasteurize milk before feeding?	Yes[1]	N: 2 (9.09)<br>E: 12 (17.39)<br>S: 0 (0.0)	N: 4 (2.48)<br>E: 10 (9.90)<br>S: 0 (0.0)	N: 178 (97.27)<br>E: 166 (97.65)<br>S: 103 (99.04)						

Variable	Category	MAP-positive herds n (%)	MAP-negative herds n (%)	Herds with information available n (%)	OR	LCL	UCL	p	AFe	AFp
	No	N: 20 (90.91)<br>E: 57 (82.61)<br>S: 3 (100)	N: 152 (94.41)<br>E: 87 (86.14)<br>S: 100 (99.01)		N: 0.26<br>E: 0.55	N: 0.05<br>E: 0.22	N: 1.53<br>E: 1.35	N: 0.14<br>E: 0.19	N: -1.87<br>E: -0.38	N: -1.70<br>E: -0.31
What do you feed your calves during the first two weeks of life?	Milk replacer[1]	N: 4 (18.18)<br>E: 12 (17.39)<br>S: 0 (0.0)	N: 36 (22.36)<br>E: 18 (17.82)<br>S: 5 (4.95)	N: 182 (99.45)<br>E: 170 (100)<br>S: 104 (100)				N: 0.84[a]<br>E: 0.96[a]		
	Milk	N: 12 (54.55)<br>E: 42 (60.87)<br>S: 2 (66.67)	N: 88 (54.66)<br>E: 63 (62.38)<br>S: 71 (70.30)		N: 1.23<br>E: 1.0	N: 0.37<br>E: 0.44	N: 4.06<br>E: 2.29	N: 1.0<br>E: 0.86	N: 0.17<br>E: 0.00	N: 0.13<br>E: 0.00
	Other	N: 6 (27.27)<br>E: 15 (21.74)<br>S: 1 (33.33)	N: 36 (22.36)<br>E: 20 (19.80)<br>S: 25 (24.75)		N: 1.5<br>E: 1.13	N: 0.39<br>E: 0.42	N: 5.77<br>E: 3.03	N: 0.57<br>E: 0.77	N: 0.30<br>E: 0.07	N: 0.18<br>E: 0.04
What do you feed your calves from three weeks on?	Milk replacer[1]	N: 11 (50.00)<br>E: 43 (62.32)<br>S: 0 (0.0)	N: 96 (59.63)<br>E: 61 (60.40)<br>S: 23 (22.77)	N: 183 (100)<br>E: 170 (100)<br>S: 104 (100)				N: 0.29[a]<br>E: 0.86[a]		
	Milk	N: 4 (18.18)<br>E: 21 (30.43)<br>S: 2 (66.67)	N: 35 (21.74)<br>E: 27 (26.73)<br>S: 55 (54.46)		N: 1.0<br>E: 1.1	N: 0.3<br>E: 0.55	N: 3.34<br>E: 2.2	N: 0.24<br>E: 0.95	N: 0.00<br>E: 0.05	N: 0.00<br>E: 0.02
	Other	N: 5 (22.73)<br>E: 5 (7.25)<br>S: 1 (33.33)	N: 27 (16.77)<br>E: 11 (10.89)<br>S: 23 (22.77)		N: 1.62<br>E: 0.65	N: 0.52<br>E: 0.21	N: 5.05<br>E: 1.99	N: 0.86<br>E: 0.96	N: 0.34<br>E: -0.32	N: 0.11<br>E: -0.03
	Is fed by the cow (suckling calves)	N: 2 (9.09)<br>E: 0 (0.0)<br>S: 0 (0.0)	N: 3 (1.86)<br>E: 2 (1.98)<br>S: 0 (0.0)		N: 5.82<br>E: <0.001	N: 0.88<br>E:<0.001	N: 38.7<br>E: >999.9	N: 0.09<br>E: 0.96	N: 0.74<br>E: n.p.	N: 0.11<br>E: -0.02
In case of bucket drinking: Does each calf have its own bucket during the first two weeks of life?	Yes[1]	N: 16 (72.73)<br>E: 46 (66.67)<br>S: 3 (100)	N: 90 (55.90)<br>E: 56 (55.45)<br>S: 47 (46.53)	N: 177<br>E: 160<br>S: 99						
	No	N: 5 (22.73)<br>E: 18 (26.09)<br>S: 0 (0)	N: 66 (40.99)<br>E: 34 (33.66)<br>S: 49 (48.51)		N: 0.43<br>E: 0.65	N: 0.15<br>E: 0.32	N: 1.22<br>E: 1.29	N: 0.11<br>E: 0.21	N: -1.14<br>E: -0.30	N: -0.27<br>E: -0.09

Variable	Category	MAP-positive herds n (%)	MAP-negative herds n (%)	Herds with information available n (%)	OR	LCL	UCL	p	AFe	AFp
In case of bucket drinking: Does each calf from three weeks on have its own bucket?	Yes[1]	N: 2 (9.09)<br>E: 9 (13.04)<br>S: 2 (66.67)	N: 16 (9.94)<br>E: 13 (12.87)<br>S: 27 (26.73)	N: 105<br>E: 58<br>S: 88						
	No	N: 10 (45.45)<br>E: 18 (26.09)<br>S: 0 (0)	N: 77 (47.83)<br>E: 18 (17.82)<br>S: 59 (58.42)		N: 1.04<br>E: 1.44	N: 0.21<br>E: 0.49	N: 5.2<br>E: 4.22	N: 0.96<br>E: 0.5	N: 0.03<br>E: 0.18	N: 0.03<br>E: 0.12
In case of bucket drinking/automatic feeding: How often are the buckets and teats cleaned for calves up to two weeks?	After every feeding[1]	N: 7 (31.82)<br>E: 27 (39.13)<br>S: 1 (33.33)	N: 93 (57.76)<br>E: 57 (56.44)<br>S: 83 (82.18)	N: 175<br>E: 162<br>S: 102				N: 0.11[a]<br>E: 0.13[a]		
	Once a day	N: 7 (31.82)<br>E: 17 (24.64)<br>S: 1 (33.33)	N: 23 (14.29)<br>E: 16 (15.84)<br>S: 7 (6.93)		N: 4.04<br>E: 2.24	N: 1.29<br>E: 0.99	N: 12.68<br>E: 5.1	N: 0.17<br>E: 0.37	N: 0.70<br>E: 0.38	N: 0.35<br>E: 0.15
	Less than once a day	N: 6 (27.27)<br>E: 15 (21.74)<br>S: 1 (33.33)	N: 32 (19.88)<br>E: 14 (13.86)<br>S: 6 (5.94)		N: 2.49<br>E: 2.26	N: 0.78<br>E: 0.96	N: 7.96<br>E: 5.35	N: 0.76<br>E: 0.37	N: 0.56<br>E: 0.38	N: 0.26<br>E: 0.14
	No cleaning	N: 1 (4.55)<br>E: 7 (10.14)<br>S: 0 (0.0)	N: 6 (3.73)<br>E: 9 (8.91)<br>S: 3 (2.97)		N: 2.2<br>E: 1.64	N: 0.23<br>E: 0.55	N: 21.05<br>E: 4.88	N: 0.98<br>E: 0.93	N: 0.51<br>E: 0.27	N: 0.06<br>E: 0.05
In case of bucket drinking/automatic feeding: How often are the buckets and teats cleaned for calves from three weeks on?	After every feeding[1]	N: 3 (13.64)<br>E: 10 (14.49)<br>S: 0 (0.0)	N: 51 (31.68)<br>E: 29 (28.71)<br>S: 73 (72.28)	N: 137<br>E: 143<br>S: 100				N: 0.22[a]<br>E: 0.06[a]		
	Once a day	N: 7 (31.82)<br>E: 23 (33.33)<br>S: 2 (66.67)	N: 26 (16.15)<br>E: 19 (18.81)<br>S: 7 (6.93)		N: 4.58<br>E: 3.51	N: 1.09<br>E: 1.37	N: 19.17<br>E: 9.0	N: 0.15<br>E: 0.06	N: 0.74<br>E: 0.53	N: 0.52<br>E: 0.37
	Less than once a day	N: 5 (22.73)<br>E: 19 (27.54)<br>S: 1 (33.33)	N: 33 (20.50)<br>E: 20 (19.80)<br>S: 11 (10.89)		N: 2.58<br>E: 2.76	N: 0.58<br>E: 1.06	N: 11.50<br>E: 7.16	N: 0.96<br>E: 0.32	N: 0.58<br>E: 0.47	N: 0.36<br>E: 0.31
	No cleaning	N: 2 (9.09)<br>E: 9 (13.04)<br>S: 0 (0.0)	N: 10 (6.21)<br>E: 14 (13.86)<br>S: 6 (5.94)		N: 3.4<br>E: 1.86	N: 0.5<br>E: 0.62	N: 23.03<br>E: 5.62	N: 0.63<br>E: 0.78	N: 0.67<br>E: 0.34	N: 0.27<br>E: 0.16

[1]= Reference category
N= Study region north
E= Study region east
S= Study region south
OR= Odds Ratio
LCL= Lower Confidence Limit
UCL= Upper Confidence Limit
[a]= global p-value
AFe= Attributable fraction exposed
AFp= Population attributable fraction
n.p.= calculation not possible
*[1] includes at least weaned or six-month-old cattle (until first calving)
*[2]calves up to two weeks
† includes only cleaning of calf pens; movement of calf pens, cleaning of calf pens, keeping calf pens empty, disinfection of calf pens, other (in combination and as not mentioned below)
‡ includes: only movement of calf pens, only keeping calf pens empty, only disinfection of calf pens, only keeping calf pens empty and movement of calf pens, only disinfection and keeping calf pens empty, only movement of calf pens and keeping calf pens empty and disinfected
